# Supplementary material for: In vitro larvicidal activity of ivermectin and povidone-iodine against Oestrus ovis
Source: PLoS One. 2021 Oct 26;16(10):e0259044. doi: 10.1371/journal.pone.0259044 (PMC8547687; doi:10.1371/journal.pone.0259044)
Supplement: Attachment — Submitted filename: Response to Reviewers.docx [file pone.0259044.s001.docx]

**Ref.: Manuscript PONE-D-21-22404, entitled “*In vitro larvicidal activity of ivermectin and povidone-iodine against Oestrus ovis*”**

**Response to Reviewers**

**Journal requirements**

- Please ensure that your manuscript meets PLOS ONE's style requirements, including those for file naming

*Reply: done*

- In your Methods section, please provide additional details regarding the ivermectin used in your study and ensure you have described the source.

*Reply: additional details regarding the ivermectin used in our study have been provided.*

**Reviewer #1**

- The results described in the manuscript are preliminary, however they are very interesting and provide the basis for clinical studies. The only negative point, but not an impediment to publication, is that the authors should have performed the in vitro assays with a greater number of repetitions.

*Reply: All assays were performed in six replicates, not in triplicate. Triplicate was a misprint*

**Reviewer #2**

- More detail is needed to for the reader to accurately interpret the design used for the study. It is noted that “Each Petri dish was seeded with 25 larvae, filled with 1 mL of each study drug” and that all assays were conducted in triplicate. I may have misunderstood, but to me this suggests that 5 treatments + saline control x 25 larvae x 3 replicates = 450 larvae were used

*Reply: We wish to thank Reviewer #2 for highlighting our mistake. Indeed, there was a misprint. All assays were performed in six replicates, not in triplicate (5 treatments + saline control x 25 larvae x 6 replicates = ~900 larvae were used)*

- It is noted that larvae for the study were collected over a number of days and that in most cases the experiments were conducted on the day of collection, but in some instances when all heads could not be processed, they were held overnight and processed the following day. Often such closely associated parasites die quickly once separated from their host, or their host dies. Thus how the replicates/treatments were established with respect to time of collection needs to be specified to ensure there is no confounding between treatment effects and time of collection.

*Reply: Only three sheep heads were held overnight in specific containers at 39°C and processed early in the morning the following day. This procedure did not significantly affect larval viability. All the nasal boots were examined by stereomicroscope. All the viable L1 larvae found were collected and transferred onto separate Petri dishes containing mucosal tissue obtained from the nasal boots.*

- Figure 1.

As I understand it Figure 1, Graph A contains a plot of all treatments, Graph B is exactly the same as A but with 3 of the treatments from Graph A omitted and Graphs C and D just contain subsets of Graph A replotted with a different scale on the X axis?. That is, for example the same data 1% ivermectin in BSSS treatment is replotted 3 times in Graphs A, B, and C. It is unusual to replot the same data repeatedly and is potentially confusing for the reader. Consider just using Graph A with statements about which treatments were significantly different in the caption.

*Reply: Figure 1 has been changed according to Reviewer #2 suggestions.*

-References.

As noted above there is at least one previous reported study of the use of ivermectin to treat human ocular myiasis (Kumar et al. 2013 Annals of Tropical Medicine and Public Health 6: 315-316) as well as a quite a number of previous reports of the effectiveness of ivermectin against Oestrus ovis larvae (in human hosts and in sheep). None of these papers have been cited in the reference list. These papers would seem to be very relevant to the current study and at least some of them should be noted.

*Reply: the study by Kumar et al. has been quoted (Reference 8) and additional references have been added (References 11, 12 and 13).*

*The following comment has been included in the Discussion: “There is already at least one paper indicating efficacy of oral ivermectin (12 mg in a single dose) in treating human conjunctival myiasis caused by O. ovis larvae.^8^ However, the use of systemic ivermectin for the management of ocular surface myiasis is rather questionable, as a topical approach with ointment and/or eye-drops would be much more appropriate.”*
