# Supplementary material for: In vitro larvicidal activity of ivermectin and povidone-iodine against Oestrus ovis
Source: PLoS One. 2021 Oct 26;16(10):e0259044. doi: 10.1371/journal.pone.0259044 (PMC8547687; doi:10.1371/journal.pone.0259044)
Supplement: Attachment — Submitted filename: Response to Reviewers BIS.docx [file pone.0259044.s002.docx]

**Ref.: Manuscript PONE-D-21-22404R1, entitled “*In vitro larvicidal activity of ivermectin and povidone-iodine against Oestrus ovis*”**

**Response to Reviewers**

**Journal requirements**

Please review your reference list to ensure that it is complete and correct. If you have cited papers that have been retracted, please include the rationale for doing so in the manuscript text, or remove these references and replace them with relevant current references. Any changes to the reference list should be mentioned in the rebuttal letter that accompanies your revised manuscript. If you need to cite a retracted article, indicate the article’s retracted status in the References list and also include a citation and full reference for the retraction notice.

*Reply: As far as we know, there are no retracted references in the list. Reference 8 (Kumar MA, Joseph NM, Srikanth K, Stephen S. Conjunctival Myiais caused by Oestrus ovis in a medical college student which responded to Ivermectin. Ann Trop Med Public Health 2013;6: 315-316), whose inclusion in the list was recommended by Reviewer #2, does not appear in PubMed, but can be retrieved in Scopus*

**Reviewer #2**

Abstract
Lines 39-41 “Kaplan-Meyer analysis
…disclosed that the survival curves were significantly lower in the samples treated with ivermectin 41 1% solution, ivermectin 1% solution + 0.6% PVP-I, and propylene glycol.” Please state lower than what. Lower than the RSSS control or lower than the other treatments
*Reply: the sentence has been changed as follows: Kaplan-Meyer analysis disclosed that the survival curves were significantly lower in samples treated with ivermectin 1% solution, ivermectin 1% solution + 0.6% PVP-I, and propylene glycol than in samples receiving other treatments or BSSS.*

Introduction
Line 54. In entomological protocol it is most common, though not essential to state both the Order and the Family e.g. [Diptera:Oestridae]

*Reply: changed according to Reviewer suggestion*

Line 115- The BSS would usually be considered a negative control. A positive control would usually mean a treatment known to be effective in killing larvae. I suggest just delete “Positive”: ie Control Petri dishes received 1 mL of BSSS.

*Reply: changed according to Reviewer suggestion*

Line 124 – Log rank test, rather than “..long rank test”

*Reply: changed according to Reviewer suggestion*

Line 147 – Again, as above, significantly lower than what?

*Reply: the sentence has been changed as follows: The survival curves were significantly lower in the samples treated with ivermectin 1% solution in propylene glycol, ivermectin 1% solution in propylene glycol + 0.6% PVP-I, and propylene glycol than in the samples receiving other treatments or BSSS.*

Lines 175-177. This is an important point which would benefit from a supporting reference.

*Reply: as recommended the following reference (#15) has been included in the list: Laing R, Gillan V, Devaney E. Ivermectin - Old Drug, New Tricks? Trends Parasitol. 2017;33: 463-472.*

Line 193 to 195 Delete “also”. That is: ‘Surprisingly enough, propylene glycol, the solubilizer chosen for ivermectin, had similar larvicidal activity.

*Reply: changed according to Reviewer suggestion*

Lines 203-204 Suggest “Our results suggest that ivermectin 1% solution in propylene glycol, ivermectin 1% solution in propylene glycol + 0.6% PVP-I, and propylene glycol might be potential candidates for the topical treatment, as ointment and/or eye-drops, for external ophthalmomyiasis caused by O. ovis.”

*Reply: changed according to Reviewer suggestion*

Line 224 – closing sentence. Suggest ‘… to establish whether or not these formulations may be considered….’ Rather than “…they..”

*Reply: changed according to Reviewer suggestion*
